# Research and knowledge transfer priorities in developmental coordination disorder: Results from consultations with multiple stakeholders

**DOI:** 10.1111/hex.12947

**Published:** 2019-08-14

**Authors:** Chantal Camden, Sabah Meziane, Désirée Maltais, Noémi Cantin, Marie Brossard‐Racine, Jade Berbari, Mélanie Couture

**Affiliations:** ^1^ School of Rehabilitation Université de Sherbrooke Research Centre from the CR CHUS Sherbrooke Québec Canada; ^2^ University of Montreal Montreal Quebec Canada; ^3^ University Laval Quebec Quebec Canada; ^4^ Université du Québec à Trois‐Rivières Trois‐Rivières Quebec Canada; ^5^ Université McGill Montreal Quebec Canada

**Keywords:** children with disabilities, engagement in research, integrated knowledge translation, priority‐setting, rehabilitation

## Abstract

**Background:**

Priority‐setting is a way to focus research and knowledge translation (KT) efforts for community‐based research partnerships (CBRP).

**Objective:**

To identify the developmental coordination disorder (DCD) research and KT priorities of stakeholders in Quebec, Canada, and their perceptions regarding the implementation of a CBRP.

**Design:**

An advisory committee oversaw the research process including an online survey and four community forums.

**Setting and participants:**

The survey was posted online and four community forums were organized. Participants included parents of children with DCD, adults with DCD, health professionals and school staff.

**Main variables:**

Stakeholder generated research and KT priorities, and optimal CBPR conditions.

**Outcome measures:**

Participants selected their top five priorities based on a predefined list of 16 research and 12 KT priorities determined in collaboration with the advisory committee. They also rated the importance of various CBRP conditions. Preliminary survey results were discussed during the forums.

**Results:**

Survey participants (n = 395) identified interwoven research and KT priorities where access to services was considered to be essential: supporting children at school; improving DCD identification and diagnosis; preventing secondary consequences; improving the organization of services and implementing effective services. Forum participants (n = 52) confirmed the relevance of these priorities and supported the establishment of a CBRP inclusive of all stakeholders to improve DCD services, research and KT.

**Discussion and conclusions:**

A general consensus emerged among all groups, but adults with DCD were more concerned with employment than were the other stakeholder groups. These findings are presently being used to shape an ongoing, online CBRP.

## 1 INTRODUCTION

Stakeholder engagement in paediatric rehabilitation research, defined as the involvement in the research process of patients and individuals without a traditional research background (eg, families of children with disabilities, children and youth with disabilities, clinicians, health‐care managers, and policy makers), have been suggested as a strategy to foster evidence uptake in clinical practice.^1^, ^2^, ^3^ Such stakeholder engagement could decrease the research‐to‐practice gap by leading to the development of more clinically relevant research questions,^4^, ^5^ and therefore more meaningful and readily applicable research results.^1^, ^6^ However, it is challenging to achieve meaningful involvement of these stakeholders in the generation of research questions and grant proposals.^1^, ^2^, ^5^ Involving the stakeholders in the identification research priorities within a given clinical domain is a strategy that has been used by some rehabilitation researchers to ensure future researches meet patients and stakeholders expectations.^7^, ^8^ For instance, in the field of cerebral palsy, McIntyre and colleagues^8^ conducted a Delphi survey of consumers, clinicians and researchers. Their study results informed the development of research priorities in cerebral palsy for a funding agency. However, it is unclear if this process supported the ongoing involvement of the stakeholders in the agency's subsequent research projects.

Community‐based research partnerships (CBRP) are reported to foster the ongoing engagement of all stakeholders throughout the research process, from the identification of research questions to the dissemination of results.^2^ Best practice principles in CBRP include ensuring that (a) roles are clearly defined, (b) responsibilities are shared, (c) there is an appropriate timeframe, (d) open communication channels are established and maintained, and (e) community needs and contexts are responded to in a timely manner.^9^, ^10^ However, there is very little evidence on how to implement these partnerships in paediatric rehabilitation. Moreover, if CBRP aim to produce new knowledge and disseminate current knowledge, more information is required about how to identify knowledge gaps and knowledge transfer (KT) priorities. One of the rare examples of KT priority setting to identify knowledge gaps in paediatric rehabilitation is provided by Russell and colleagues,^9^ who surveyed researchers as part of their organizational strategic planning. Similar processes should be replicated with more stakeholder groups, and within specific fields of paediatric rehabilitation, to help identify where KT efforts should be focused to further decrease the knowledge‐to‐practice gap. Health‐care conditions for which there are important knowledge‐to‐practice gaps and a need for raising community awareness about the condition might particularly benefit from developing CBRP, since CBRP facilitate the sharing of knowledge to reduce health discrepancies.^11^

Developmental coordination disorder (DCD), a condition characterized by coordination difficulties that develop early on and impact on children's daily lives,^12^ is an area where a CBRP could be particularly beneficial. It is well recognized that there is a general lack of awareness about the condition,^13^, ^14^ and that there is inequitable access to the services.^13^ These services are however required to address the issues these individuals face such as academic difficulties, lower quality of life and poor peer relationships.^16^, ^17^ Best practices for DCD stress the need for collaborative work and capacity building to increase equitable access to services and raise community awareness.^13^, ^18^ Community‐based research partnerships could thus well support the implementation of DCD best practices by bringing together different stakeholders to advocate and design interventions aiming at increasing community awareness and access to services. In the light of the lack of information in the scientific literature regarding the development of a DCD CBRP, it would seem prudent to begin the partnership by identifying the DCD priorities for KT and for research, based on different stakeholders’ perspectives, and to explore stakeholders’ interest for engaging in a research partnership.

This paper thus presents the results of a study conducted to identify research and KT priorities in DCD in Quebec, Canada. Stakeholders’ perceptions regarding the development of a CBRP are also presented.

## 2 METHOD

### 2.1 Study design

This descriptive study was grounded in community‐based participatory research^11^ and used an integrated knowledge translation (iKT) approach to involve stakeholders throughout the research process and to foster the implementation of study results.^15^ The study was approved by the ethics committee of the CR CHUS, and all study participants provided informed consent.

### 2.2 Population

The targeted population for this study were DCD stakeholders living in the province of Quebec, Canada at the time of the study. Anyone above 18 years old having an interest in DCD was eligible to participate. Participants were categorized into one or more stakeholder groups: parents of a child with DCD, adults with DCD, health‐care and education professionals, and ‘others’ such as community‐based stakeholders (eg day care staff, sport coaches) and researchers. Research participants were recruited via social media, an email campaign and word‐of‐mouth, in collaboration with an advisory committee and study partners (the provincial DCD association and four rehabilitation and health‐care centres).

### 2.3 Study procedures and data collection

An advisory committee was convened to oversee the research process, from study design to iKT. There were clear selection criteria established for this committee, and potential candidates were interviewed to determine if they met the selection criteria. The aim was to have a committee that represented a wide variety of stakeholder groups. Candidates were thus questioned regarding their interests, experience and expertise, and their desire to contribute to advancing DCD research and KT. Financial compensation guidelines were also established for the committee. Recruitment procedures for the advisory committee members aimed to reach and provide equal and fair engagement opportunities to as many people as possible (as opposed to selecting stakeholders known by the research team).^2^ Thus social media, an email campaign and word‐of‐mouth were used. The recruitment campaign was conducted in collaboration with the project partners (ie, the provincial DCD parent association and four rehabilitation centres). In all, nine stakeholders contacted the research team (three parents, including one studying to become a special education teacher, one young adult with suspected DCD, and four clinicians including two involved with the parent association and one completing a graduate degree). They were all interviewed. Since they all met the selection criteria, represented different perspectives and were from different regions, they were all invited to join the advisory committee. All agreed to do so. The activities of the advisory committee took place during online committee meetings, and via email exchanges. Eight online meetings were organized during the 12‐month duration of the study. During the kickoff meeting, committee members were provided with a short training session about clinical research and were invited to discuss and finalize the objectives and procedures governing the committee. Subsequent meetings focused on the attainment of research objectives: reviewing the study design and data collection tools, discussing recruitment strategies, interpreting results and participating in long‐term iKT and partnership activities. Members who did not attend a meeting were provided with the materials via email and their input was requested for the various research objectives. Some members attended all meetings, while other only responded only to email requests.

The advisory committee reviewed the online survey to be sent to DCD stakeholders to ask them to select their research and KT priorities. This survey was based on a study conducted to identify research priorities in cerebral palsy in Australia.^8^ One of the authors, the project's principal investigator (CC) contacted the Australian team to access the complete list of proposed themes that had been generated by the cerebral palsy stakeholders. Meetings with the research team and the advisory committee were organized to review each of these potential themes for relevance to DCD and for clarity in the present study's provincial context. They were also encouraged to explore if new priorities should be included. The committee members engaged in a consensus process which resulted in the identification of 16 research priorities and 12 KT priorities. These priorities were proposed in the online survey, where stakeholders were asked to select their top five research and KT priorities among the ones proposed.

The online survey was circulated to potential participants online via social media, the advisory committee and project partners. In addition to the research and KT questions, participants were also asked to rate the importance of optimal CBRP conditions. These conditions were modified from those of Wallerstein and colleagues.^11^ A Likert Scale (from 1 – not at all important to 7 – very important) was used for the ratings. Open‐ended questions allowed participants to list other priorities and partnership conditions, and to justify their selections.

Four community forums, defined as open assemblies where everyone having an interest in a particular topic is invited,^16^ were organized in different cities across the province, approximately one month after the survey was launched. Preliminary survey findings (n = 308) were presented and discussed in small groups moderated by a member of the research team. At the end of the forum, participants were asked to rate their satisfaction with the forum and the extent to which the forum met their expectations. A 5‐point Likert scale was used and forum participants could provide additional comments. Members of the research team acted as note takers during the forums to ensure key comments were captured.

### 2.4 Statistical analysis

Descriptive statistics were used to describe survey participants and satisfaction with forums. As recommended,^17^, ^18^ research and KT priority rankings were scored using a regular‐interval scale (scores of 5, 4, 3, 2, 1) or an incremental interval scale wherein regular‐interval values were squared (scores of 25, 15, 9, 4, 1). Both scales yielded similar results, but the incremental interval scale was adopted as it better reflected differences between priorities. Mean priority scores (and standard deviations) were calculated, and the top five priorities were identified for the entire sample, and by stakeholder subgroup. Means and standard deviations were also used to assess the importance of CBRP conditions.

A thematic analysis^19^ was performed on the open‐ended survey questions and forum field notes to understand reasons underlying the participants’ selection of research and KT priorities, as well as their perception of the optimal conditions required for the CBRP. The quoted comments presented below were freely translated from French.

## 3 RESULTS

### 3.1 Survey

Table 1 presents the sociodemographic characteristics of the 395 participants who completed the online survey. The most common stakeholder group was “parent of a child with DCD” (44%). Most parents reported that their child was under the age of 12 (89%), had a formal diagnosis of DCD (96%) and had at least one other neurodevelopmental disorder (65%). In contrast, 33% of ‘adults with DCD’ had a formal DCD diagnosis. Some participants self‐identified as being members of more than one stakeholder group (eg 15% of parents were also education professionals). Most ‘healthcare’ and ‘education’ professionals were occupational therapists (63%) and teachers (60%), respectively.

**Table 1:** Sociodemographic characteristics of survey participants (n = 395)

	N (%)
Parents/caregiver of a child with DCD	174 (44%)
Mother*/*female	165 (96%)
Have only one child with DCD	148 (85%)
Child with DCD aged 5‐12 y old	154 (89%)
Child has a formal medical diagnostic of DCD	167 (96%)
Child has a diagnosis of another neurodevelopmental disorder	113 (65%)
Most common comorbidity: ADHD	76 (67%)
Child is currently receiving rehabilitation services	158 (91%)
Most common service: Occupational therapy	138 (87%)
Adults with DCD	12 (3%)
Female	9 (75%)
Have a formal medical diagnosis of DCD	4 (33%)
Have had the diagnosis for over 5 y	2 (50%)
Have a diagnosis of another neurodevelopmental disorder	7 (58%)
Most common comorbidities:	
Learning disability (including dyslexia, dysorthographia)	3 (25%)
ADHD	2 (17%)
Currently working	6 (50%)
Currently at school	6 (50%)
Health‐care professional	*138 (35%)*
Female	135 (98%)
Have 2‐7 y of experience	60 (44%)
Work as an occupational therapist	87 (63%)
Work mostly in a rehabilitation centre	62 (45%)
Provide services to children with DCD	120 (87%)
Education professional	*106 (27%)*
Female	102 (96%)
Have <2 y of experience	34 (40%)
Work as a regular teacher	64 (60%)
Work with children with DCD	86 (81%)
Other participants	14 (4%)

Tables 2 and 3 present the findings regarding research and KT priorities, respectively. Supporting children's success at school and improving the identification, screening and diagnosis of DCD were, respectively, ranked first and second, both as research and KT priorities. These two priorities were among the top five for almost all stakeholder groups. The third, fourth and fifth research priorities pertained to the prevention of the secondary consequences of DCD, the organization of health and education services, and the effectiveness of rehabilitation interventions. Slight variations existed in the priorities of the different stakeholder groups. For instance, only adults with DCD included integrating the labour market as one of their priorities.

**Table 2:** Research priorities of survey participants

	Scores using an incremental interval scale (SD)					
	All stakeholders	Parents	Adults with DCD	Health stakeholders	Education stakeholders	Others
**What are the best interventions to foster success at school for children with DCD?**	**5.74 (8.84)^1^**	**7.07 (9.24)^1^**	0.58 (1.16)	**5.84 (8.58)^3^**	**8.27 (10.18)^1^**	**3.50 (7.55)^5^**
**How can we improve the identification, screening and diagnosis of people with DCD?**	**4.28 (8.36)^2^**	**4.20 (8.21)^5^**	**10.75 (11.55)^1^**	**6.80 (9.92)^1^**	**4.26 (8.40)^2^**	2.14 (4.72)
**How can we prevent the secondary consequences of DCD (self‐esteem problems, anxiety, obesity, etc)?**	**3.75 (7.68)^3^**	**5.38 (8.93)^2^**	**9.67 (10.87)^2^**	3.23 (6.79)	**3.34 (7.28)^4^**	**3.93 (7.66)^4^**
**How can we organize health and education systems to improve services for people with DCD and their family ?**	**3.61 (7.26)^4^**	**4.54 (7.84)^4^**	2.42 (7.20)	**4.46 (7.96)^4^**	**3.94 (7.21)^3^**	**7.43 (10.58)^1^**
**What are the best rehabilitation interventions?**	**3.11 (6.45)^5^**	2.18 (4.72)	0.92 (2.57)	**6.60 (8.86)^2^**	1.67 (4.87)	**4.21 (6.24)^3^**
How can we improve quality of life for people with DCD?	2.85 (6.74)	**4.98 (8.41)^3^**	0.42 (1.16)	2.23 (6.25)	1.84 (4.88)	**6.07 (10.53)^2^**
What is the potential of cerebral reorganization to decrease difficulties experienced by people with DCD?	2.49 (6.40)	3.55 (7.72)	**2.75 (5.03)^5^**	3.29 (7.03)	1.66 (5.05)	2.79 (6.87)
How can we best equip parents so that they have the tools to better support their children with DCD?	2.29 (5.29)	2.07 (5.04)	2.42 (5.07)	**4.29 (6.89)^5^**	1.67 (4.87)	0.79 (2.39)
What are the difficulties experienced by children with DCD?	1.48 (4.43)	1.93 (4.76)	2.08 (5.09)	0.64 (2.67)	**2.67 (6.08)^5^**	0.29 (1.07)
How can we decrease the service gaps experienced by individuals with DCD?	1.47 (4.78)	1.50 (4.61)	2.67 (6.23)	2.22 (5.96)	1.72 (5.36)	3.29 (5.94)
How can we support people with DCD and their families during the different periods of their life?	1.16 (3.96)	1.43 (4.32)	1.50 (3.50)	1.22 (3.97)	1.20 (4.26)	2.57 (5.79)
How can people with DCD be more prepared for the labour market?	1.03 (3.53)	1.55 (4.33)	**2.92 (7.42)^4^**	1.22 (3.89)	0.60 (2.64)	0.21 (0.43)
What is the optimal therapy intensity for people with DCD?	0.91 (3.52)	0.36 (2.33)	1.33 (4.62)	2.38 (5.45)	0.51 (2.20)	1.79 (6.68)
What are the best strategies to raise awareness of DCD among the general population?	0.90 (3.43)	1.55 (4.39)	2.00 (3.64)	1.08 (3.86)	0.36 (1.85)	0.29 (1.07)
What are the service obstacles for people with DCD?	0.45 (2.72)	0.69 (3.46)	**3.42 (8.21)^3^**	0.59 (3.04)	0.23 (1.64)	0.00 (0.00)
How can age, gender et others health problems influence the effectiveness of interventions?	0.25 (2.01)	0.03 (0.31)	0.00 (0.00)	0.81 (3.60)	0.047 (0.40)	0.00 (0.00)

Bold values indicates the top‐5 priorities for each stakeholder's groups.

**Table 3:** Knowledge transfer priorities of survey participants

	Scores using an incremental interval scale (SD)					
	All stakeholders	Parents	Adults with DCD	Health stakeholders	Education stakeholders	Others
**Interventions promoting success at school**	**4.92 (8.19)^1^**	**2.99 (3.96)^1^**	1.67 (3.45)	**6.80 (8.89)^2^**	**4.76 (8.29)^1^**	**3.86 (7.69)^2^**
**Strategies for identifying, screening and diagnosing DCD**	**3.68 (8.40)^2^**	1.52 (3.32)	**11.25 (12.39)^1^**	**6.93 (10.67)^1^**	**3.04 (7.66)^4^**	1.86 (6.67)
**Resources and strategies supporting school professionals**	**3.27 (6.36)^3^**	**2.07 (3.20)^2^**	1.50 (4.58)	**4.36 (6.72)^4^**	**4.45 (7.67)^2^**	**3.14 (5.27)^5^**
**Strategies preventing the secondary consequences of DCD**	**2.83 (6.87)^4^**	**1.72 (3.27)^4^**	**7.17 (8.91)^2^**	2.94 (6.60)	**2.24 (6.08)^5^**	**5.93 (9.89)^1^**
**Strategies to support teachers**	**2.79 (6.21)^5^**	**1.90 (3.04)^3^**	0.50 (1.17)	3.04 (5.88)	**4.23 (7.87)^3^**	1.57 (4.29)
Strategies to support parents	2.71 (6.26)	1.26 (2.41)	0.67 (1.56)	**5.89 (8.87)^3^**	1.81 (5.12)	2.79 (6.87)
Difficulties experienced by children with DCD at school	2.71 (6.36)	**1.68 (3.14)^5^**	**4.50 (8.17)^4^**	**3.16 (6.52)^5^**	2.02 (5.07)	**3.64 (9.05)^3^**
Strategies to support the quality of life of individuals with DCD	1.77 (5.28)	1.06 (2.57)	1.00 (1.81)	2.59 (6.16)	1.55 (5.28)	1.93 (3.83)
Strategies improving social participation (eg at school, at work, during sports and leisure time)	1.47 (4.39)	0.79 (2.00)	1.75 (4.63)	2.57 (5.86)	1.25 (3.58)	**3.50 (7.55)^4^**
Strategies raising awareness about DCD among the general population	1.41 (4.64)	0.99 (2.43)	**5.67 (8.19)^3^**	2.07 (5.81)	0.40 (2.00)	2.86 (5.75)
Strategies to support the parent's ability to manage their children's DCD	1.37 (4.26)	1.08 (2.57)	0.83 (2.59)	1.86 (4.51)	1.60 (5.12)	0.36 (1.08)
Strategies to support labour market integration	0.72 (3.09)	0.52 (1.62)	**3.42 (8.21)^5^**	1.15 (3.93)	0.30 (1.54)	0.00 (0.00)

Bold values indicates the top‐5 priorities for each stakeholder's groups.

In the open‐ended survey questions, most new priorities were closely related to the above‐mentioned top five priorities (eg, studying the impact of using a computer instead of hand writing at school; evaluating the impact of educational intervention plans). Newly identified priorities related entirely to better understanding and managing DCD comorbidities (eg, sleep and visual problems, learning disabilities).

The participants’ justification of their priorities suggested that they selected priorities based on their current situation and needs. They did, however, acknowledge that priorities might change over time.

School was difficult for that child, tiring, because his teachers did not know about DCD, we did not get help, and others’ lack of awareness converted this condition into a disability. My priorities are grounded in the difficulties we have to face on a daily basis. [Healthcare professional]

I do not currently think about employment, but I am sure I will worry about that in the near future. [Parent]

The priority justifications also shed light on how the top five priorities were closely interwoven. Identifying children with DCD was key to accessing services; and timely access to effective health, rehabilitation and educational services was key to supporting children and their families at school and preventing secondary consequences.

First, we need to identify children with DCD. Then, to improve support at school. I think school and health services need to be better coordinated [Parent and healthcare professional]

Early intervention can help. However few services are offered. The consequences of this lack of intervention became obvious at school [Parent and educational professional]

Increasing DCD awareness was clearly the most important KT priority. Participants sought KT strategies to increase their own knowledge of DCD, but also to inform and empower teachers, families, physician and rehabilitation professionals, as well as society as a whole.

The lack of awareness about DCD is a big problem right now. You don’t know what to do, who to ask for help; since it is invisible, you don’t know how to explain it to others. Access to resources and help is difficult, and there are almost no services. [Parent]

If we could all have access to a set of resources, that would make things easier in a clinical setting, and help families and schools. Lots of good things are happening everywhere, but a lack of dissemination does not allow clinicians to use these resources with their clients [Healthcare professional]

### 3.2 Community forums

Fifteen parents, 30 health‐care professionals, 3 education professionals and 4 adults with DCD participated in the four community forums (n = 52). Participants confirmed the above‐mentioned priorities and justifications derived from the survey. Many community forum participants shared stories about how difficult it was to get help, and how important it was to foster positive well‐being and social experiences for individuals with DCD. Most of the discussions pertained to increasing DCD awareness and access to diagnosis and services. Many forum participants had also completed the survey. These individuals noted that while all proposed items were indeed important, their top five priorities were based on their current personal and professional needs.

Forum participant responses helped to explain why the research and KT priorities often targeted the same themes, as these responses underlined the importance of disseminating and implementing what is already known in DCD research. For instance, they were unsure of the state of the research knowledge, but felt that if well‐documented strategies and resources to share information about DCD, and interventions to best support school teachers existed, we should focus our efforts on KT. However, if we had insufficient knowledge about how best to support children and teachers at school, we should focus on creating new research knowledge. With regard to the apparent consensus between groups, one forum participant suggested to further explore priorities by comparing priority results for parents of children younger and older than 12 years, to explore if school was a priority for parents having younger children while other priorities (eg work) would be emerged for parents with older children. No significant difference was found. Forum participants also suggested keeping the survey open for an additional 2 months to potentially maximize the representativeness of survey participants. In addition, they recommended implementing recruitment strategies for the survey respondees that specifically targeted young adults with DCD and physicians. Following the forums, for example, a young adult with DCD made a video targeting young adults with DCD, and some clinician forum participants sent the survey directly to physicians with whom they collaborated. The addition of survey participants did not change the top five priorities.

Table 4 presents mean importance scores for the conditions necessary to optimally support a CBRP. There was a general consensus between stakeholder groups that open and respectful dialogue was the most important condition. Survey participants also commented on the importance of (a) including a broad range of stakeholders including health‐care and education professionals, (b) fostering knowledge exchange and opportunities for learning about DCD, and (c) using technologies to facilitate engagement with a CBRP, especially for individuals living in rural areas.

**Table 4:** Importance of conditions supporting a research‐community partnership

	Mean (SD)					
	All stakeholders	Parents	Adults	Health stakeholders	Education stakeholders	Others
The partnership needs to provide a place for open et respectful dialogue	6.03 (1.19)	5.78 (1.26)	6.56 (0.88)	6.19 (1.09)	6.28 (1.05)	5.71 (1.80)
Expectations and roles in the partnership need to be clear	5.99 (1.14)	5.76 (1.16)	5.78 (0.97)	6.12 (1.14)	6.08 (1.24)	6.00 (1.10)
People need to engage in the partnership to advance DCD research and KT, and not for personal reasons	5.92 (1.28)	5.92 (1.23)	6.56 (1.01)	5.87 (1.39)	5.92 (1.14)	6.50 (0.84)
Various engagement strategies need to be offered, according to people's interests and availabilities.	5.60 (1.32)	5.30 (1.42)	6.11 (1.35)	5.76 (1.21)	5.84 (1.28)	5.86 (0.90)
In‐person human support needs to be available to optimize participation in the partnership	5.58 (1.34)	5.58 (1.30)	5.56 (1.13)	5.46 (1.41)	5.92 (1.10)	5.33 (0.52)
Measures need to be taken to sustain the partnership over time	5.52 (1.40)	5.43 (1.30)	5.67 (1.41)	5.52 (1.49)	5.39 (1.38)	5.67 (0.52)
Material support needs to be available to optimize participation in the partnership	5.43 (1.31)	5.41 (1.22)	5.33 (1.32)	5.36 (1.38)	5.63 (1.33)	5.00 (1.00)
Financial support needs to be available to optimize participation in the partnership	5.07 (1.47)	5.06 (1.41)	3.78 (1.71)	5.13 (1.44)	5.22 (1.46)	4.17 (1.17)
People in the partnership need to come from various socio‐economic, cultural and geographic backgrounds	4.90 (1.73)	4.72 (1.81)	5.44 (1.24)	5.05 (1.57)	5.14 (1.74)	4.67 (1.97)
People in the partnership need to have a good understanding of DCD	4.75 (1.77)	5.02 (1.78)	3.67 (1.32)	4.71 (1.70)	4.46 (1.66)	4.00 (2.00)
Power and resources need to be equally distributed in the partnership	4.73 (1.44)	4.91 (1.36)	3.78 (1.20)	4.55 (1.48)	4.76 (1.42)	4.57 (1.62)
Researchers in the partnership need to be well renowned	4.53 (1.53)	4.71 (1.49)	5.00 (1.41)	4.52 (1.52)	4.40 (1.51)	3.29 (1.70)
A formal agreement needs to be signed by people and organizations participating in the partnership	4.26 (1.58)	4.41 (1.57)	3.78 (1.20)	4.17 (1.55)	4.12 (1.60)	4.17 (1.60)

During the forums, many participants asked for more concrete information about what a CBRP would look like. The research team presented a vision where an interactive website could be created. This generated many exchanges about how best to use the Internet and social media to support an CBRP.

Satisfaction with community forums was high (4.8/5) and forum participants reported that the forums met with their expectations (4.6/5), even if most voiced concerns about how an eventual CBRP could be implemented and sustained. Subsequent to the community forums, the advisory committee reviewed and approved a plan where the CBRP would focus on building the following three‐way interactive communication strategy: (a) a blog to share credible, research‐informed content https://tdcrecherche.com/, (b) an open Facebook page where anyone could contribute https://www.facebook.com/TDCrecherche/, and (c) a quarterly newsletter summarizing CBRP activities.

## 4 DISCUSSION

The main study objective was to describe the research and KT priorities of DCD stakeholders. In contrast to what is known regarding other paediatric conditions, supporting children at school was a priority for most stakeholder groups. This may be explained by the widely established lack of awareness about DCD and its consequences in schools,^14^, ^20^ and by the concurrent fact that children's difficulties (eg, play differences, academic difficulties) often first emerge at school, where DCD impact on self‐esteem and quality of life also become apparent.^21^ Furthermore, our collaborative research process resulted in a high proportion of participants being education professionals and parents. Since priorities are grounded in individuals’ daily life, it is not surprising that school was deemed a high priority.

Supporting children at school closely relates to the second priority, DCD identification and diagnosis. Our results support widely reported findings that physicians and health‐care professionals generally declare having limited knowledge about DCD.^12^, ^15^ Increasing DCD awareness could in turn increase access to services, an issue closely related to the top five priorities. Multiple calls in the scientific literature have been made to reorganize school and health services (Priority #4) in order to foster access to early intervention, and to implement response‐to‐intervention service delivery models and coordinated services.^13^, ^18^ It is also accepted that interventions should be evidence‐informed, and anchored in functional approaches,^22^ which coincides with Priority #5. Such service delivery models could prevent secondary consequences, such as poor self‐esteem^21^ (Priority #3).

Our results also showed that there was a general consensus among stakeholders for both research and KT priorities, which offers a unique opportunity for collaboration between stakeholders. This finding also differs from that of studies in other fields, such as cerebral palsy, that have reported different research priorities between stakeholders groups.^7^, ^8^ This general consensus should not hide slight but relevant subgroup differences. The most important stakeholder priorities group difference was for adults with DCD, who face challenges that are particular to their age group.^23^, ^24^ Since our results also highlight that priorities might change over time, addressing adults with DCD priorities appears crucial, since children with DCD will likely become adults with DCD. Likewise, families who identified school as a current priority may identify employment as a priority in the future. Research and KT efforts should therefore be proactive and address issues faced by different stakeholders.

Our KT and research priority findings coalesced around similar themes. We were unable to identify other published results comparing research and KT priorities in DCD or in other populations. Since themes proposed in priority‐setting research are generally broad, it is likely that specific actions might require KT efforts while others might require more research, depending on the state of the knowledge. Nevertheless, our results highlight the value of our two‐step process – survey and community forums – and of engaging with a variety of stakeholder's subgroups. Our results also demonstrate desire of our stakeholders to access scientific knowledge and engage in partnerships with researchers. The highest‐rated partnership conditions echoed previous publications, where patients want clear roles and opportunities to meaningfully engage with researchers, and to build reciprocal relationships.^5^

With regard to stakeholder's engagement in this specific research, the advisory committee was of great help regarding how to move forward with implementing the CBRP following the discussion in the forums. We worked with the committee to find strategies to more clearly define roles and expectations within the partnership, and to facilitate broad access to the information. The advantage of using social media to improve the transfer of health knowledge is already documented.^25^, ^26^, ^27^ Our goal was to use our partnership webpage as a virtual community of practice, which allows individuals with specialized interests who are geographically dispersed to collaborate.^27^ At present, posts and emails from our new CBRP have reached over 200 individuals and efforts are ongoing to maintain this partnership. However, as the CBRP is intended as a strategy for both ‘push’ (ie dissemination) and ‘pull’ (ie generating research ideas) exchanges, more efforts are needed to engage CBRP members to be more active, for example, on our Facebook page.

The main study limitations were that we collected self‐reported information for sociodemographic information and thus we could not verify that parents indeed had a child with a diagnosis of DCD. Priorities also provide a limited snapshot in time, for a given population in a given context. Priorities may change over time and might not be generalizable in other cultures, health‐care and education systems, even if the issues with regards to awareness and access to services are quite consistently reported across countries in the scientific literature. Future studies should track priorities longitudinally and replicate the process across different cultures and settings. More efforts to include stakeholder groups that were underrepresented in the present study (eg, adults with DCD and physicians) are also needed, and priorities for children with DCD also need to be explored.

## 5 CONCLUSION

This article contributes to the generation of knowledge about how to best foster patient and stakeholder engagement in research in order to ensure that research efforts are aligned with their priorities. Specifically, it illustrated a process of using different research methods, including an advisory committee, online surveys and community forums, to engage with families, clinicians, teachers, adults with DCD and researchers. This process went beyond traditional identification of research priorities as it also explored the need for knowledge transfer and opportunities to maintain collaborations through the establishment of an academic community‐based partnership. The research and KT priorities identified in this study inspire our current studies aimed at fostering school success and participation for children with DCD, and access to diagnosis and services. The partnership that is presently being established is also being used to support recruitment for DCD studies, the transfer of knowledge and information sharing with stakeholders, and to foster an ongoing conversation with stakeholders about research and KT priorities.

## CONFLICT OF INTEREST

The researchers have stated that they had no interests which might be perceived as posing a conflict or bias.

## Data Availability

The data that support the findings of this study are available from the corresponding author upon reasonable request.

## References

[1] Shen S , Doyle‐Thomas KAR , Beesley L , et al. How and why should we engage parents as co‐researchers in health research? A scoping review of current practices. Health Expect. 2017;20(4):543‐554. 27516003 10.1111/hex.12490 PMC5513005

[2] Camden C , Shikako‐Thomas K , Nguyen T , et al. Engaging stakeholders in rehabilitation research: a scoping review of strategies used in partnerships and evaluation of impacts. Disabil Rehabil. 2015;37(15):1390‐1400. 25243763 10.3109/09638288.2014.963705

[3] Morris C , Shilling V , McHugh C , Wyatt K . Why it is crucial to involve families in all stages of childhood disability research. Dev Med Child Neurol. 2011;53(8):769‐771. 21518349 10.1111/j.1469-8749.2011.03984.x

[4] Henderson J , Brownlie E , Rosenkranz S , Chaim G , Beitchman J . Integrated knowledge translation and grant development: addressing the research practice gap through stakeholder‐ informed research. J Can Acad Child Adolesc Psychiatry. 2013;22(4):268. 24223045 PMC3825466

[5] Shippee ND , Domecq Garces JP , Prutsky Lopez GJ , et al. Patient and service user engagement in research: a systematic review and synthesized framework. Health Expect. 2015;18(5):1151‐1166. 23731468 10.1111/hex.12090 PMC5060820

[6] Herschell AD , McNeil CB , McNeil DW . Clinical child psychology's progress in disseminating empirically supported treatments. Clin Psychol Sci Pract. 2004;11(3):267‐288.

[7] Griffiths KM , Jorm AF , Christensen H , Medway JO , Dear KBG . Research priorities in mental health, Part 2: an evaluation of the current research effort against stakeholders' priorities. Aust N Z J Psychiatry. 2002;36(3):327‐339. 12060181 10.1046/j.1440-1614.2001.01024.x

[8] McIntyre S , Novak I , Cusick A . Consensus research priorities for cerebral palsy: a Delphi survey of consumers, researchers, and clinicians. Dev Med Child Neurol. 2010;52(3):270‐275. 19694780 10.1111/j.1469-8749.2009.03358.x

[9] Russell DJ , McCauley D , Novak I , et al. (2015). Developing a Knowledge Translation Strategy for a Centre of Childhood Disability Research: Description of the Process. Scholarly and Research Communication. 7(1): 0105237, 11.

[10] Teal R , Moore AA , Long DG , Vines AI , Leeman J . A community‐academic partnership to plan and implement an evidence‐based lay health advisor program for promoting breast cancer screening. J Health Care Poor Underserved. 2012;23(2 suppl):109‐120. 22643559 10.1353/hpu.2012.0076 PMC12278316

[11] Wallerstein N , Duran B . Community‐based participatory research contributions to intervention research: the intersection of science and practice to improve health equity. Am J Public Health. 2010;100(suppl 1):S40‐S46. 20147663 10.2105/AJPH.2009.184036 PMC2837458

[12] Association AP . Desk Reference to the Diagnostic Criteria from DSM‐5. Arlington, VA: American Psychiatric Publishing 2014.

[13] Harris SR , Mickelson EC , Zwicker JG . Diagnosis and management of developmental coordination disorder. CMAJ. 2015;187(9):659‐665. 26009588 10.1503/cmaj.140994 PMC4467929

[14] Wilson BN , Neil K , Kamps PH , Babcock S . Awareness and knowledge of developmental co‐ordination disorder among physicians, teachers and parents. Child Care Health Dev. 2013;39(2):296‐300. 22823542 10.1111/j.1365-2214.2012.01403.x PMC3579234

[15] Canadian Institues of Health Research . Guide to Knowledge Translation Planning at CIHR: Integrated and End‐of‐grant Approaches. Ottawa, ON: CIHR; 2012.

[16] Tétreault C , Beaupré P , Marier Deschênes P , et al. Comment mobiliser la communauté grâce au forum communautaire ?Une méthode participative à la portée de l’intervenant social. Serv Social. 2013;59(2):63‐75.

[17] Mitton C , Donaldson C . Health care priority setting: principles, practice and challenges. Cost Eff Resour Alloc. 2004;2(1):3. 15104792 10.1186/1478-7547-2-3 PMC411060

[18] Ranson MK , Chopra M , Atkins S , Dal Poz MR , Bennett S . Priorities for research into human resources for health in low‐ and middle‐income countries. Bull World Health Organ. 2010;88:435‐443. 20539857 10.2471/BLT.09.066290 PMC2878144

[19] Miles MB , Huberman AM , Saldaña J . Qualitative Data Analysis: An Expanded Sourcebook. Thousand Oaks, Califorinia: Sage Publications 1994: 2.

[20] Wang T‐N , Tseng M‐H , Wilson BN , Hu F‐C . Functional performance of children with developmental coordination disorder at home and at school. Dev Med Child Neurol. 2009;51(10):817‐825. 19416344 10.1111/j.1469-8749.2009.03271.x

[21] Missiuna C , Moll S , King S , King G , Law M . A trajectory of troubles. Phys Occup Ther Pediatr. 2007;27(1):81‐101. 17298942

[22] Smits‐engelsman BCM , Blank R , Van der kaay A‐C , et al. Efficacy of interventions to improve motor performance in children with developmental coordination disorder: a combined systematic review and meta‐analysis. Dev Med Child Neurol. 2013;55(3):229‐237. 23106530 10.1111/dmcn.12008

[23] Kirby A , Edwards L , Sugden D . Emerging adulthood in developmental co‐ordination disorder: parent and young adult perspectives. Res Dev Disabil. 2011;32(4):1351‐1360. 21334175 10.1016/j.ridd.2011.01.041

[24] Gagnon‐Roy M , Jasmin E , Camden C . Social participation of teenagers and young adults with developmental co‐ordination disorder and strategies that could help them: results from a scoping review. Child Care Health Dev. 2016;42(6):840‐851. 27481762 10.1111/cch.12389

[25] Lewis B , Rush D . Experience of developing Twitter‐based communities of practice in higher education. Res Learn Technol. 2013;21:13.

[26] Dubé L , Bourhis A , Jacob RA Toward a typology of virtual communities of practice. Interdiscip J Inform Knowl Manage. 2006;1:69‐92.

[27] Evans C , Yeung E , Markoulakis R , Guilcher S . An online community of practice to support evidence‐based physiotherapy practice in manual therapy. J Contin Educ Health Prof. 2014;34(4):215‐223. 25530291 10.1002/chp.21253

